# ﻿Into the unknown: the first barcode-assisted checklist of Psocoptera (Insecta, Psocodea) of Georgia with a census on country species richness

**DOI:** 10.3897/zookeys.1168.103666

**Published:** 2023-06-27

**Authors:** Armen Seropian, Eka Arsenashvili, Natalia Bulbulashvili, Anano Shubashishvili, Giorgi Iankoshvili, Mariami Todua, Ana Ananiashvili, Shota Japarashvili, Tinatin Chkhartisvhili, Aleksi Memishishi, Sopio Balkhamishvili, Beka Chitadze, Elisabeth Karalashvili, Levan Mumladze, Nils Hein, Björn Rulik

**Affiliations:** 1 Institute of Ecology, Ilia State University, Cholokashvili av. 3/5 Tbilisi, 0162, Georgia; 2 Rustaveli st. 8, 1400, Gori, Georgia; 3 Institute of Zoology, Ilia State University, Cholokashvili av. 3/5 Tbilisi, 0162, Georgia; 4 Leibniz Institute for the Analysis of Biodiversity Change (LIB), Museum Koenig, Bonn, Germany

**Keywords:** Biodiversity, CaBOL, faunistics, South Caucasus

## Abstract

This checklist reports 47 species of Psocoptera from 15 families and three suborders from Georgia, of which 31 species are recorded for the first time, increasing the known fauna of the country by more than 65%. Of these, 37 species have been barcoded, representing 210 Barcode Identification Numbers (BINs). An additional 14 species are expected to occur in Georgia but remain undiscovered, meaning that only ≈ 77% of the fauna is currently documented. Barcodes, comments on distributions, and images of voucher specimens are given followed by a map of the sampling sites.

## ﻿Introduction

Psocoptera, known as the booklice and barklice, is an order of hemimetabolous insects having approximately 6000 described species worldwide (Anonby 2019), often regarded as the most primitive hemipteroids alive today (Lyal 1985). Despite being called “lice”, psocids are not parasites, instead they are free living, generally herbivoroues or detritivorous insects, feeding on organic debris and microflora (Lienhard 1998) and are classified into three suborders: Trogiomorpha (booklice), Troctomorpha (booklice), and Psocomorpha (barklice). While most of them (mainly species belonging to the suborder Psocomorpha) are bark- or leaf-dwellers found outdoors, there are domiciliary species exhibiting excellent powers of dispersal and readily spread by humans all over the world. For some species, this makes it difficult to reconstruct the original distribution or to distinguish between native and introduced once (Schneider 2010).

Although Psocoptera has been classified as an order for much of recent history, it was shown by Yoshizawa and Lienhard (2010) that Phthiraptera (the true lice) have evolved within the Troctomorpha suborder and, based on both morphological and molecular data, are probably sister group to Liposcelididae, or, various lines of Phthiraptera may have budded off independently in the infraorder Nanopsocetae within Troctomorpha.

Thus, to maintain monophyly, the former orders Psocoptera and Phthiraptera are now placed in the order Psocodea (Yoshizawa and Lienhard 2010). However, since psocids and true lice have quite different ecologies, and are studied by different methods, for practical reasons Psocoptera is still typically treated as a group in the traditional way, but referred to as Psocodea: ’Psocoptera’ (Anonby 2019; Golub 2016, 2019). This practical approach was also applied in the present work.

The first attempt to assess the species richness of the psocid fauna of Georgia was made by Danka (1950), who documented seven families comprising eight species from Sokhumi Botanical Garden. In her later work (Danka 1955), ten species belonging to six families have been reported from Batumi and Sochi Botanical Gardens, six of which have been reported for the first time, thus raising the number of species known from Georgia to 13. Based on the data of subsequent articles (Danka 1957, 1968), the overall number of Georgian psocids has been raised to 16 local species from ten different families combined in two suborders (Table 1). It should be noted that all studies mentioned above were carried out in the western part of Georgia near the Black Sea coast, in the territories of Adjara and Abkhazia, and since the last review of Georgian Psocoptera (Danka 1968), there has been no survey conducted on this group, hence leaving the hidden psocid diversity of most of the country unrevealed for more than half a century.

**Table 1.** Changes in composition of Georgian barklice fauna through time and different surveys.

Taxon name	Number of species from Georgia reported within the study/known by the end of the study				
	Danka 1950	Danka 1955	Danka 1957	Danka 1968	Current study
	8	14/16	16/16	16/16	45/47
**Suborder Psocomorpha**					
**Amphipsocidae**					
*Kolbiaquisquiliarum*					+
**Caeciliusidae**					
*Caeciliusfuscopterus*					+
*Valenzuelaatricornis*					+
*Valenzuelaburmeisteri*	+		+	+	+
*Valenzuelaflavidus*		+	+	+	+
*Valenzuelapiceus*		+	+	+	+
**Ectopsocidae**					
*Ectopsocopsiscryptomeriae*					+
*Ectopsocusbriggsi*	+	+	+	+	+
*Ectopsocusmeridionalis*					+
*Ectopsocusvishnyakovae*					+
**Elipsocidae**					
*Elipsocushyalinus*					+
*Elipsocusmoebiusi*					+
*Hemineurahispanica*					+
**Epipsocidae**					
*Bertkauialucifuga*					+
**Lachesillidae**					
*Lachesillabernardi*					+
*Lachesillapedicularia*					+
*Lachesillaquercus*			+	+	+
**Mesopsocidae**					
*Mesopsocuslaticeps*					+
*Mesopsocusunipunctatus*	+		+	+	
**Peripsocidae**					
*Peripsocusalboguttatus*		+	+	+	+
*Peripsocusdidymus*					+
*Peripsocusgolubae*					+
*Peripsocusphaeopterus*		+	+	+	+
*Peripsocussubfasciatus*	+	+	+	+	+
**Philotarsidae**					
*Aaroniellabadonneli*	+	+	+	+	+
*Philotarsuspicicornis*	+		+	+	+
**Psocidae**					
*Amphigerontiacontaminata*	+		+	+	+
*Loensiafasciata*					+
*Loensiavariegata*					+
*Neopsocusrhenanus*					+
*Metylophorusnebulosus*					+
*Psococerastisgibbosa*					+
*Psocusbipunctatus*					+
*Trichadenotecnumalexanderae*					+
*Trichadenotecnumsexpunctatum*					+
**Stenopsocidae**					
*Enderleinellaobsoleta*					+
*Graphopsocuscruciatus*		+	+	+	+
*Stenopsocusimmaculatus*		+	+	+	+
**Trichopsocidae**					
*Trichopsocusdalii*	+	+	+	+	+
**Suborder Troctomorpha**					
**Liposcelididae**					
*Embidopsocusenderleini*					+
*Liposcelisdecolor*			+	+	
*Liposcelismeridionalis*					+
*Liposcelisrufa*					+
**Suborder Trogiomorpha**					
**Psyllipsocidae**					
*Dorypteryxdomestica*					+
*Psyllipsocusramburii*					+
**Trogiidae**					
*Cerobasisguestfalica*					+
*Lepinotusreticulatus*					+

In the present contribution, we provide the results of recent countrywide investigations and significantly improve the existing knowledge on the psocid diversity of Georgia.

## ﻿Materials and methods

### ﻿Locations and methods

**Figure 1.**
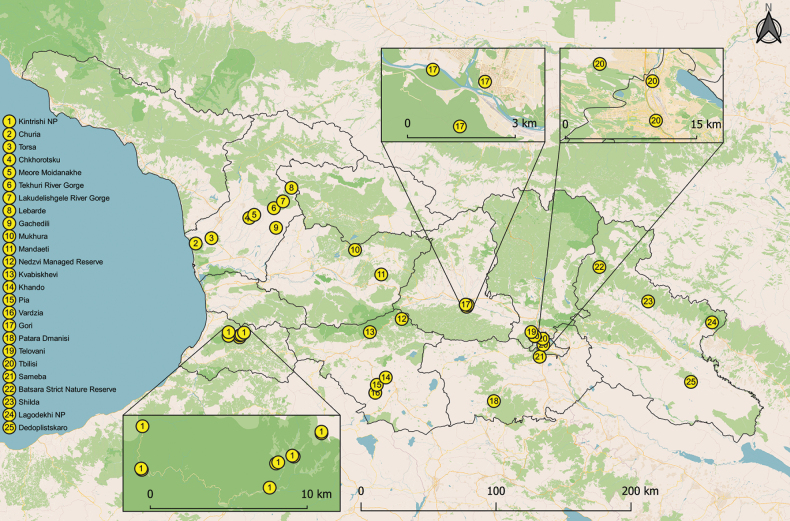
Map of the barklice sampling sites in Georgia within the CaBOL project 2018–2022.

The main part of the studied material was collected within the framework of Caucasus Barcode of Life (CaBOL) project, being the most ambitious arthropod inventories ever performed in Georgia by the members of the GGBC (Georgian-German Biodiversity Center) and the CaBOL team of the Institute of Ecology, Ilia State University (https://ggbc.eu/). Most of the samples were collected using 18 Malaise traps installed in various habitats of the Kintrishi National Park from 20 April to 3 November 2018 at altitudes ranging from 403 m up to 2465 m (Thormann et al. 2019), and the one-year Malaise trap program, with traps (one per location) installed in Tbilisi city, Mandaeti (Chiatura municipality) and Shilda (Kvareli municipality) villages as part of the Urban Biodiversity Research project. Additional material was collected during short field trips and expeditions to Dedoplistskaro (21 April 2021), Sameba (Kumisi, 29 September 2021), Samtskhe-Javakheti (08–11 October 2021, 10–14 October 2022), Batsara-Babaneuri Strict Nature Reserve (27–30 May 2022), Telovani (9 July 2022), Lagodekhi National Park (18–20 July 2022), Samegrelo-Zemo Svaneti (28 July – 03 August 2022), Gori (25 May, 12–14 August, 06 September, 24 October, 05 November 2022), Patara Dmanisi (25 October 2022), Torsa (Khobi municipality, 16–27 August 2022), Mukhura (Tkibuli municipality, 16–26 August 2022) and Dighomi (Tbilisi, 22 October, 19 November 2022) villages, via malaise traps, aspirators, hand collecting, and beating methods. Details for sampling locations are given in Fig. 1 and in the Suppl. material 1.

Collected specimens were preserved in 96% ethanol, stored in a freezer at -22 °C at the scientific collections of Ilia State University. Unless otherwise stated, all material has been determined by the corresponding author, using a Zeiss Stemi Stereo Microscope with 8:1 Zoom and a Zeiss Apo 1.5× FWD 53 mm front lens attached and keys, provided by Lienhard (1998, 2006) and Saville (2008). For the classification of psocids, we followed Johnson et al. (2022).

Photographs of preserved specimens were taken using a Canon EOS 60D camera with a Canon MP-E 65mm f/2.8 1–5× Macro Photo Lens mounted on a Novoflex Castel-L Focusing Rack. Digital images were prepared using Zerene Stacker image stacking software and Adobe Photoshop CS6.

### ﻿DNA processing

DNA was extracted from whole samples using the Quick-DNA Magbead Plus Kit (Zymo Research). Partial sequences of cytochrome oxidase subunit I (COI) were amplified by polymerase chain reaction (PCR) using the primer pair LCOI490-JJ and HCO2198-JJ (Astrin and Stüben 2008). Thermal conditions included denaturation at 95 °C for 1 min, followed by first cycle set (15 cycles): 94 °C for 30 s, annealing at 55 °C for 1 min (–1 °C per cycle) and extension at 72 °C for 1:30 min. Second cycles set (25 cycles): 94 °C for 35 s, 45 °C for 1 min, 72 °C for 1:30 min, followed by 1 cycle at 72 °C for 3 min and final extension step at 72 °C for 5 min. PCR amplicons were visualized on 1% agarose gels using 1.7 μl of PCR product. Sequencing of the unpurified PCR products in both directions was conducted at the Beijing Genomics Institute (Hong Kong, CN) by using the amplification primers. Sequence analysis was performed using Geneious Prime 2022.1.1 (http://www.geneious.com). Extracted DNA was deposited in the scientific collections of Ilia State University, Tbilisi, Georgia and aliquots will be deposited at LIB Biobank at Museum Koenig, Bonn, Germany, while the sequences have been submitted to Barcode of Life Data System (BOLD) databases. The newly obtained DNA barcodes of COI sequences were checked out against the BOLD systems database (http://www.boldsystems.org/index.php). Barcode Index Number (BIN) (Ratnasingham and Hebert 2013) for the sequenced taxa and for their nearest neighbor in BOLD systems (if they had a BIN) are also given. For the calculation of sequence differentiation, we used p distance as performed in the BOLD system.

## ﻿Results

**Figure 2.**
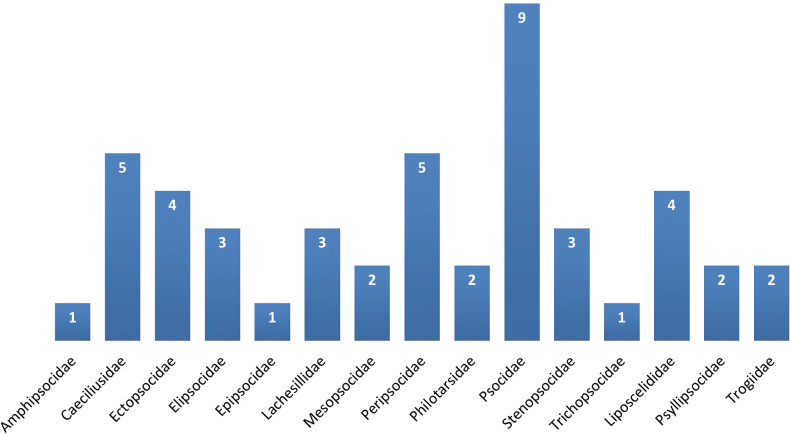
Species ratio within the families of the Georgian barklice (*n* = 47).

In total 2353 specimens were collected representing 45 species comprising 15 families (Fig. 2). New country records are marked with an asterisk (*) and detailed data on the collected material is provided in Suppl. material 1. From the collected material, 371 barklice specimens were submitted for barcoding pipeline and only 210 quality barcodes (658 bp length barcodes, with no stop codons, indels or deletions) representing 37 species were generated so far. Barcode information is given under each barcoded species listed below.

### ﻿Annotated checklist of Georgian Psocoptera

**Suborder Psocomorpha Badonnel, 1951**

Family *Amphipsocidae Pearman, 1936

**Note.** Representatives of the family have not been previously known to occur in Georgia. One species has been recorded within the current study.

#### ﻿Genus *Kolbia Bertkau, 1882

* ***K.quisquiliarum* Bertkau, 1882**

Fig. 3A

**Figure 3.**
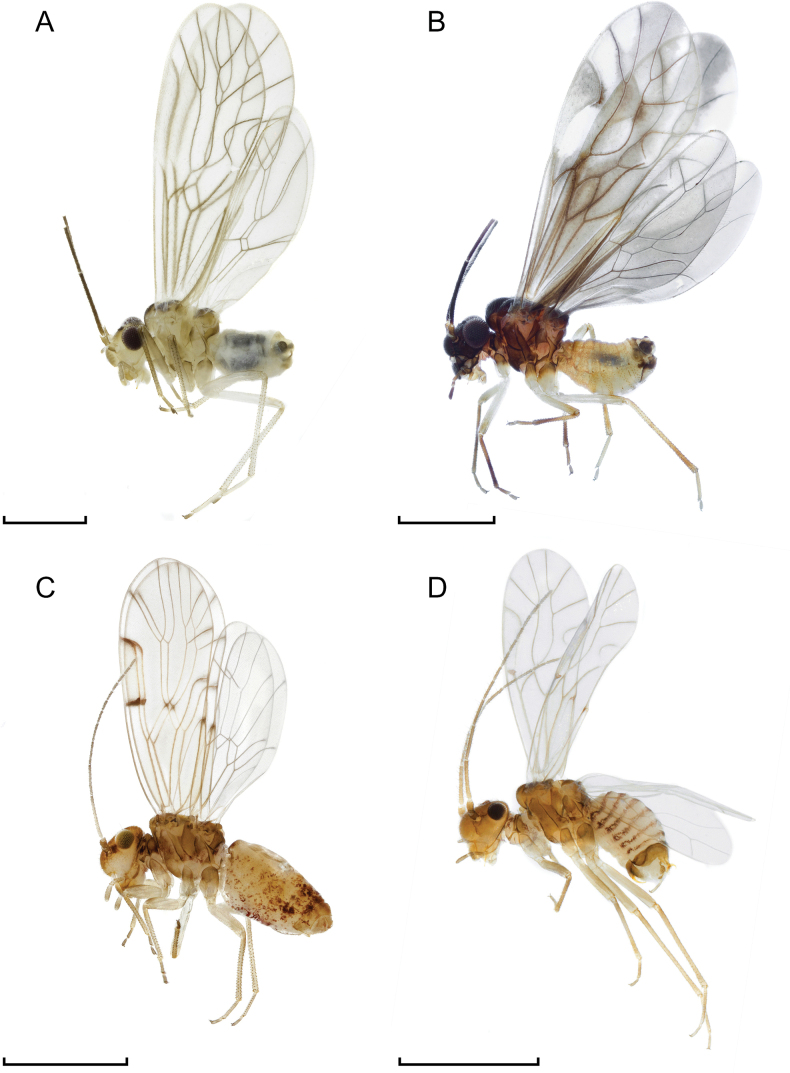
General habitus in lateral view of **A***Kolbiaquisquiliarum* Bertkau, 1882, male **B***Caeciliusfuscopterus* (Latreille, 1799), male **C***Ectopsocusbriggsi* McLachlan, 1899, female **D***Lachesillapedicularia* (Linnaeus, 1758), male. Scale bars: 1 mm.

**Material examined.** 91 ♂♂.

**Distribution in Georgia.** Kintrishi National Park.

**Distribution in Caucasus.** Georgia.

**Global distribution.** European part of Western Palaearctic (Lienhard 2016).

**Genetics.** We obtained eleven barcodes from the specimens with CaBOL-IDs 1027555, 1027556, 1027557 (BOLD:AFC5937), 1027571, 1027572, 1012880, 1012881, 1012882, 1012883, 1012884 and 1012885 (BOLD:AFC5936) (maximum p-distance 1.52%) with the nearest neighbor in BOLD systems being *K.quisquiliarum* from Finland (BOLD:ACG2800, mean p-distance 7.2%). Examined specimens perfectly matched the description provided by Lienhard (1998). The large COI distance (over 5%) between the populations of *K.quisquiliarum* might indicate the existence of a cryptic species complex in western Palaearctic, and further study is needed to solve this puzzle.

**Remarks.** The species is characterized by micropterous females.

**Family Caeciliusidae Kolbe, 1880**

**Note.** Five species are recorded from Georgia.

#### ﻿Genus *Caecilius Curtis, 1837

* ***C.fuscopterus* (Latreille, 1799)**

Fig. 3B

**Material examined.** 74 ♀♀, 60 ♂♂.

**Distribution in Georgia.** Kintrishi National Park, Lagodekhi National Park, Shilda.

**Distribution in Caucasus.** Karachay-Cherkessia (Golub 2016), Armenia (Golub 2019), Georgia.

**Global distribution.** Palaearctic (Lienhard 2016).

**Genetics.** Eleven barcodes were obtained from the specimens with CaBOL-IDs 1012650, 1012720, 1012721, 1012722, 1012893, 1012895, 1012896, 1027603, 1027607, 1027611, 1027613 (BOLD:AEJ9302, maximum p-distance 1.56%) originating from two populations, with the nearest neighbor in BOLD systems being *C.fuscopterus* from Finland (BOLD:ACG3628, mean p-distance 5.43%). Examined specimens perfectly matched the description provided by Lienhard (1998) (e.g., wing venation and pattern). Given the large COI distance (over 5%) between the populations of *C.fuscopterus* might indicate the existence of a cryptic species complex in western Palaearctic, and further study is needed to solve this puzzle.

#### ﻿Genus *Valenzuela* Navas, 1924

* ***V.atricornis* (McLachlan, 1869)**

**Material examined.** 42 ♀♀, 64 ♂♂.

**Distribution in Georgia.** Churia, Kintrishi National Park, Meore Moidanakhe, Shilda.

**Distribution in Caucasus.** Georgia.

**Global distribution.** Holarctic (Lienhard 2016).

**Genetics.** We obtained three identical barcodes from the specimens with CaBOL-IDs 1026997, 1026998 and 1033176 (BOLD:ACG3154) that were identical to *V.atricornis* from Finland (BOLD:ACG3154).

**Remarks.** Originally *V.atricornis* is a species with Palaearctic distribution, introduced to North America from Hungary (Mockford 1993).

***V.burmeisteri* (Brauer, 1876)**

**Material examined.** 20 ♀♀, 9 ♂♂.

**Distribution in Georgia.** Sokhumi Botanical Garden (Danka 1950, 1957, 1968), Kintrishi National Park, Qvabiskhevi, Shilda.

**Distribution in Caucasus.** Karachay-Cherkessia (Golub 2016), Armenia (Golub 2019), Georgia (Danka 1950, 1957, 1968).

**Global distribution.** Holarctic (Lienhard 2016).

**Genetics.** A single barcode was obtained from the specimen with CaBOL-ID 1027647 (BOLD:AEX9052) with the nearest neighbor in BOLD systems being *V.burmeisteri* from Germany (BOLD:AEX9052, p-distance 0.46%).

***V.flavidus* (Stephens, 1836)**

**Material examined.** 152 ♀♀.

**Distribution in Georgia.** Batumi Botanical Garden (Danka 1955, 1957, 1968), Churia, Lagodekhi National Park, Kintrishi National Park, Shilda, Tbilisi.

**Distribution in Caucasus.** Karachay-Cherkessia (Golub 2016), Armenia (Golub 2019), Georgia (Danka 1955, 1957, 1968; as *Caeciliusflavidus* Stephens, 1836).

**Global distribution.** Holarctic (Lienhard 2016).

**Genetics.** Nine barcodes were obtained from specimens with CaBOL-IDs 1012664, 1012665, 1012666, 1012667, 1012668, 1012694, 1012695, 1026989, 1026990 (BOLD:AAN8447, maximum p-distance 0.46%), with the nearest neighbor in BOLD systems being *V.flavidus* from Canada (BOLD:AAN8447, mean p-distance 0.12%).

**Remarks.** The species is known to have asexual parthenogenetic populations in Europe, consisting of triploid females, but is believed to have sexual and asexual populations in North America as well (de Moya 2022).

***V.piceus* (Kolbe, 1882)**

**Material examined.** 1 ♀.

**Distribution in Georgia.** Batumi Botanical Garden (Danka 1955, 1957, 1968), Tbilisi.

**Distribution in Caucasus.** Karachay-Cherkessia (Golub 2016), Georgia (Danka 1955, 1957, 1968; as *Caeciliuspiceus* Kolbe, 1882).

**Global distribution.** European part of Western Palaearctic (Lienhard 2016).

**Family Ectopsocidae Roesler, 1940**

**Note.** Four species are recorded from Georgia.

#### ﻿Genus *Ectopsocopsis Badonnel, 1955

* ***E.cryptomeriae* (Enderlein, 1907)**

**Material examined.** 258 ♀♀, 168 ♂♂.

**Distribution in Georgia.** Chkhorotsku, Dedoplistskaro, Kintrishi National Park, Mandaeti, Shilda, Tbilisi.

**Distribution in Caucasus.** North Caucasus: Krasnodar Krai (Danka 1955, 1968); Georgia.

**Global distribution.** Cosmopolitan (Lienhard 2016).

**Genetics.** We obtained eleven nearly identical barcodes from the specimens with CaBOL-IDs 1012684, 1012918, 1027620, 1027621, 1027623, 1027624, 1030883, 1030884, 1032266, 1032267, 1032270 (BOLD:AAN8449, maximum p-distance 0.31%) with the nearest neighbor in BOLD systems being *E.cryptomeriae* from Canada (BOLD:AAN8449, maximum p-distance 0.16%).

**Remarks.***Ectopsocopsiscryptomeriae* is a species of an Asian origin (Schneider 2010), with the first European record from Sochi Botanical Garden as *Ectopsocuslepnevae* Danka, 1955.

#### ﻿Genus *Ectopsocus* McLachlan, 1899

***E.briggsi* McLachlan, 1899**

Fig. 3C

**Material examined.** 156 ♀♀, 175 ♂♂.

**Distribution in Georgia.** Sokhumi Botanical Garden (Danka 1950, 1957, 1968), Batumi Botanical Garden (Danka 1955, 1957, 1968), Chkhorotsku, Churia, Dedoplistskaro, Gori, Lagodekhi National Park, Kintrishi National Park, Mandaeti, Patara Dmanisi, Shilda, Tbilisi.

**Distribution in Caucasus.** Armenia (Golub 2019), Georgia.

**Global distribution.** Cosmopolitan (Lienhard 2016).

**Genetics.** We obtained six barcodes from the specimens with CaBOL-IDs 1012678, 1026993, 1027564, 1027565, 1027609, 1032269 (BOLD:AAN8452, maximum p-distance 0.76%) identical to COI of *E.briggsi* from United States and Canada (BOLD:AAN8452) in BOLD systems.

* ***E.meridionalis* Ribaga, 1904**

**Material examined.** 11 ♀♀.

**Distribution in Georgia.** Patara Dmanisi, Tbilisi.

**Global distribution.** Subcosmopolitan - has not been recorded from Australia (Lienhard 2016).

**Genetics.** We obtained six barcodes from the specimens with CaBOL-IDs 1027569, 1027570 (BOLD:ADB3092), 1032232, 1032233, 1032234, 1032235 (BOLD:AAM8931) (mean p-distance 2.53%) identical to COI of *E.meridionalis* from Costa Rica, Germany (BOLD:ADB3092) and Canada (BOLD:AAM8931) in BOLD systems.

**Remarks.** Cryptogenic species of unknown origin (Schneider 2010).

* ***E.vishnyakovae* Schmidt, 1993**

**Material examined.** 69 ♀♀ (macropterous), 1 ♀ (brachypterous).

**Distribution in Georgia.** Dighomi village, Tbilisi.

**Distribution in Caucasus.** Armenia (Svadjan et al. 1963; Golub 2019), Georgia.

**Global distribution.** Armenia, Turkmenistan (Lienhard 2016), Iran (Khandehroo et al. 2015), Georgia.

**Genetics.** We obtained six identical barcodes from the specimens with CaBOL-IDs 1027578, 1027579, 1027580, 1027625, 1027642, 1027643 (BOLD:AEJ8025). There are no barcodes of the species available in BOLD systems as we submit the first ones.

**Remark.** This is by far the northernmost record of the species after it was reported from Armenia (Yerevan Botanical Garden) as *E.brunneus* Vishnyakova, 1963 (Svadjan et al. 1963), extending its known distribution by 160 km north.

Family *Elipsocidae Kolbe, 1882

**Note.** The representatives of the family have not previously been known to occur in Georgia. Three species have been recorded within the current study.

#### ﻿Genus *Elipsocus Hagen, 1866

* ***E.hyalinus* (Stephens, 1836)**

**Material examined.** 17 ♀♀.

**Distribution in Georgia.** Kintrishi National Park, Mukhura, Tbilisi.

**Distribution in Caucasus.** Georgia.

**Global distribution.** Palaearctic (Lienhard 2016).

**Genetics.** We obtained a single barcode from the specimen with CaBOL-ID 1032265 (BOLD:AFD9323). There are no barcodes of the species available in BOLD systems as we submit the first one.

* ***E.moebiusi* Tetens, 1891**

**Material examined.** 12 ♀♀, 12 ♂♂.

**Distribution in Georgia.** Kintrishi National Park.

**Distribution in Caucasus.** Karachay-Cherkessia (Golub 2016), Armenia (Golub 2019), Georgia.

**Global distribution.** Western Palaearctic; Canada (Lienhard 2016).

**Genetics.** We obtained nine barcodes from the specimens with CaBOL-IDs 1027594, 1027595, 1027596, 1027598, 1027626, 1027627, 1027628, 1027629, 1027630 (BOLD:AFD0492, maximum p-distance 0.46%) with the nearest neighbor in BOLD systems being *E.moebiusi* from Canada (BOLD:ACK6397, mean p-distance 4.26%).

#### ﻿Genus *Hemineura Tetens, 1891

* ***H.hispanica* (Enderlein, 1907)**

**Material examined.** 1 ♀.

**Distribution in Georgia.** Pia (Samtskhe-Javakheti).

**Distribution in Caucasus.** Armenia (Svadjan et al. 1963; Danka 1968; Golub 2019), Georgia.

**Global distribution.** South Caucasus; Southern Europe (Lienhard 2016).

**Genetics.** We obtained a single barcode from the specimen with CaBOL-ID 1020306 (BOLD:AEV8540). There are no barcodes of the species available in BOLD systems as we submit the first one.

Family *Epipsocidae Karny, 1930

**Note.** The representatives of the family have not previously been known to occur in Georgia. One species has been recorded within the current study.

#### ﻿Genus *Bertkauia Kolbe, 1882

* ***B.lucifuga* (Rambur, 1842)**

**Material examined.** 2 ♀♀.

**Distribution in Georgia.** Tekhuri River gorge (Martvili).

**Distribution in Caucasus.** Georgia.

**Global distribution.** Western Palaearctic (Lienhard 2016).

**Remarks**. Species with mainly parthenogenetic reproduction and apterous females. Only few reports of males are known (Lienhard 1998).

**Family Lachesillidae Karny, 1930**

**Note.** Three species are recorded from Georgia.

#### ﻿Genus *Lachesilla* Westwood, 1840

* ***L.bernardi* Badonnel, 1938**

**Material examined.** 38 ♂♂.

**Distribution in Georgia.** Mandaeti, Shilda, Tbilisi.

**Distribution in Caucasus.** Georgia.

**Global distribution.** Western Palaearctic (Lienhard 2016).

**Genetics.** There are no barcodes of the species available in BOLD systems as we submit the first ones. We obtained seven nearly identical barcodes from the specimen with CaBOL-IDs 1012906, 1027584, 1027586, 1027587, 1027588, 1027591, 1027592 (BOLD:ACA3100, maximum p-distance 0.46%).

* ***L.pedicularia* (Linnaeus, 1758)**

Fig. 3D

**Material examined.** 30 ♀♀, 32 ♂♂.

**Distribution in Georgia.** Kintrishi National Park, Mandaeti, Mukhura, Shilda, Tbilisi.

**Distribution in Caucasus.** Armenia (Svadjan et al. 1963; Danka 1968; Golub 2019), Georgia.

**Global distribution.** Cosmopolitan (Lienhard 2016).

**Genetics.** We obtained twelve barcodes from the specimens with CaBOL-IDs 1012698, 1012910, 1012911, 1027583, 1027585, 1027615, 1027616, 1027617, 1027645, 1032262, 1032263, 1032264 (BOLD:AAF1729, maximum p-distance 0.91%) with the nearest neighbor in BOLD systems being *L.pedicularia* from Canada (BOLD:AAN8449, maximum p-distance 0.46%).

***L.quercus* (Kolbe, 1880)**

Fig. 3B

**Material examined.** 26 ♀♀, 64 ♂♂.

**Distribution in Georgia.**Danka 1957, 1968; Shilda, Tbilisi.

**Distribution in Caucasus.** Armenia (Svadjan et al. 1963; Golub 2019), North Caucasus: Krasnodar Krai (Danka 1955, 1968; Golub 2016), Karachay-Cherkessia (Golub 2016); Georgia.

**Global distribution.** Trans-Palaearctic (Lienhard 2016).

**Genetics.** We obtained six barcodes from the specimens with CaBOL-IDs 1027568, 1027599, 1027600, 1027601, 1027602, 1027619 (BOLD:ADD4145, maximum p-distance 1.06%) with the nearest neighbor in BOLD systems being *L.quercus* from Finland (BOLD:ADD4145, mean p-distance 0.86%).

**Family Mesopsocidae Enderlein, 1901**

**Note.** Two species are recorded from Georgia.

#### ﻿Genus *Mesopsocus* Kolbe, 1880

* ***M.laticeps* (Kolbe, 1880)**

**Material examined.** 1 ♂.

**Distribution in Georgia.** Gori.

**Distribution in Caucasus.** Georgia.

**Global distribution.** Holarctic (Lienhard 2016).

***M.unipunctatus* Kolbe, 1880**

**Distribution in Georgia.** Sokhumi Botanical Garden (Danka 1950, 1957, 1968); Shilda.

**Distribution in Caucasus.** Armenia (Svadjan et al. 1963; Danka 1968; Golub 2019), Georgia (Danka 1950, 1957, 1968), North Caucasus: Karachay-Cherkessia (Golub 2016).

**Global distribution.** Holarctic (Lienhard 2016).

**Family Peripsocidae Roesler, 1944**

**Note.** Four species are recorded from Georgia.

#### ﻿Genus *Peripsocus* Hagen, 1866

***P.alboguttatus* (Dalman, 1823)**

Fig. 4A

**Figure 4.**
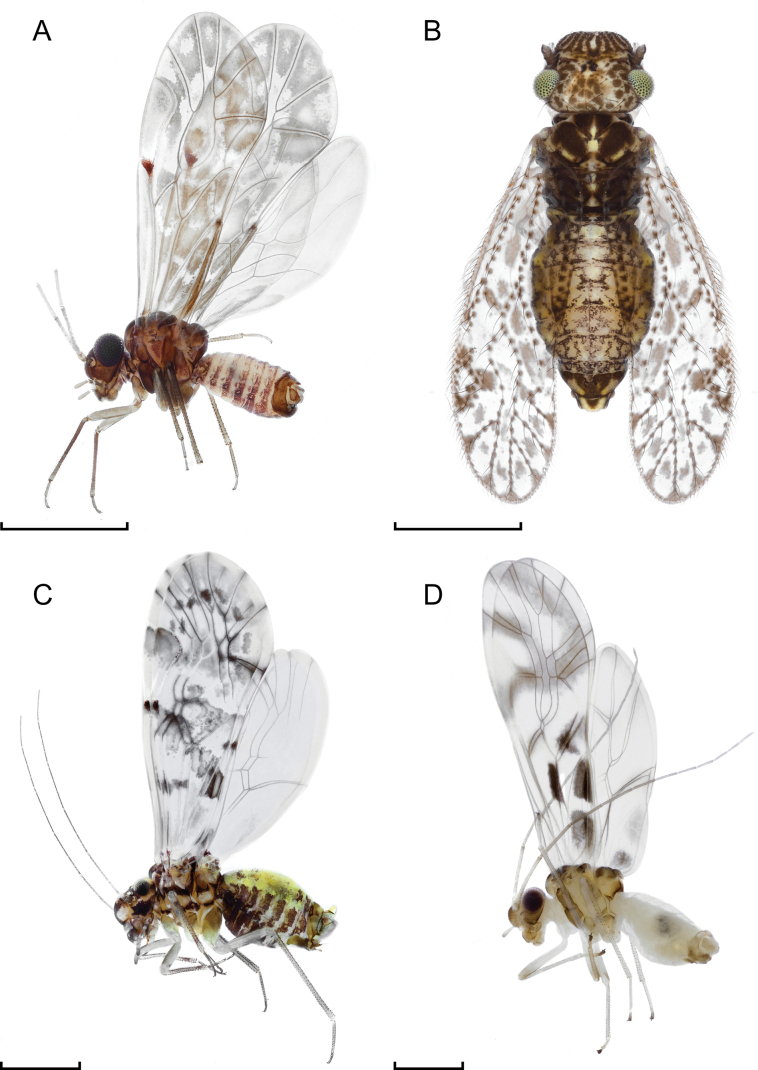
General habitus of **A***Peripsocusalboguttatus* (Dalman, 1823), male, lateral view **B***Aaroniellabadonneli* (Danka, 1950), female, dorsal view **C***Trichadenotecnumsexpunctatum* (Linnaeus, 1768), male, lateral view **D***Graphopsocuscruciatus* (Linnaeus, 1768), male, lateral view. Scale bars: 1 mm.

**Material examined.** 5 ♀♀, 7 ♂♂.

**Distribution in Georgia.** Batumi Botanical Garden (Danka 1955, 1957, 1968), Kintrishi National Park, Mandaeti, Mukhura, Nedzvi Managed Reserve.

**Distribution in Caucasus.** Georgia (Danka 1955), North Caucasus: Karachay-Cherkessia (Golub 2016).

**Global distribution.** Holarctic (Lienhard 2016).

**Genetics.** Five nearly identical barcodes were obtained from specimens with CaBOL-IDs 1012699, 1012700, 1012701, 1012702 and 1012891 (BOLD:ACN1571, maximum p-distance 0.46%), with the nearest neighbor in BOLD systems *P.alboguttatus* from Bulgaria (BOLD:AAF3894, maximum p-distance 0.46%).

* ***P.didymus* Roesler, 1939**

**Material examined.** 2 ♀♀, 5 ♂♂.

**Distribution in Georgia.** Churia, Kintrishi National Park.

**Distribution in Caucasus.** Georgia.

**Global distribution.** Palaearctic (Lienhard 2016).

* ***P.golubae* Lienhard, 2006**

**Material examined.** 3 ♀♀, 1 ♂.

**Distribution in Georgia.** Kintrishi National Park.

**Distribution in Caucasus.** Georgia.

**Global distribution.** Caucasus (Lienhard 2006).

**Genetics.** Four identical barcodes from the specimens with CaBOL-IDs 1027573, 1027574, 1027593, 1027612 (BOLD:AEJ8129) are the first ones of the species that we submit in BOLD systems.

**Remarks.** Described from southern Russia (Krasnodar Krai) this is the first time the species is reported from South Caucasus, extending its known range south by 190 km.

***P.phaeopterus* (Stephens, 1836)**

**Material examined.** 3 ♀♀, 2 ♂♂.

**Distribution in Georgia.** Batumi Botanical Garden (Danka 1955, 1957, 1968), Grigoleti, Lagodekhi National Park, Mukhura.

**Distribution in Caucasus.** Armenia (Golub 2019), Georgia (Danka 1955, 1957, 1968), North Caucasus: Karachay-Cherkessia (Golub 2016).

**Global distribution.** Holarctic (Lienhard 2016).

***P.subfasciatus* (Rambur, 1842)**

**Material examined.** 62 ♀♀.

**Distribution in Georgia.** Sokhumi Botanical Garden (Danka 1950, 1957, 1968), Batumi Botanical Garden (Danka 1955, 1957, 1968), Churia, Kintrishi National Park.

**Distribution in Caucasus.** Georgia (Danka 1950, 1955, 1957, 1968), North Caucasus: Karachay-Cherkessia (Golub 2016).

**Global distribution.** Holarctic (Lienhard 2016).

**Genetics.** Sixteen barcodes were obtained from specimens with CaBOL-IDs 1012659, 1012660, 1012661, 1012662, 1012877, 1026994, 1030871, 1030872, 1030873, 1030874, 1030875, 1030876, 1030877, 1030879, 1012723, 1012724 (BOLD:AAN8450), all identical to each other and to COI of *P.subfasciatus* from Germany (BOLD:AAN8450).

**Family Philotarsidae Pearman, 1936**

**Note.** Two species are recorded from Georgia.

#### ﻿Genus *Aaroniella* Mockford, 1951

***A.badonneli* (Danka, 1950)**

Fig. 4B

**Material examined.** 22 ♀♀.

**Distribution in Georgia.** Sokhumi Botanical Garden (Danka 1950, 1957, 1968), Batumi Botanical Garden (Danka 1955, 1957, 1968), Gachedili, Kintrishi National Park.

**Distribution in Caucasus.** Georgia (Danka 1950, 1955, 1957, 1968; as *Philotarsusbadonneli* Danka, 1950), North Caucasus: Krasnodar Krai (Danka 1955, 1968).

**Global distribution.** Trans-Palaearctic (Lienhard 2016).

**Genetics.** We obtained five nearly identical barcodes from the specimens with CaBOL-IDs 1027632, 1027633, 1027635, 1027636, 1027697 (BOLD:AAN8445, maximum p-distance 0.15%) with the nearest neighbor in BOLD systems being *Aaroniella* sp. from United States (BOLD:AAN8445, maximum p-distance 0.15%).

**Remarks.***Aaroniellabadonneli* is the only psocid species originally described from Georgia (Danka 1950). It was named after a French entomologist Andre Badonnel, who specialized in Psocoptera. No males of the species are known to be found.

#### ﻿Genus *Philotarsus* Mockford, 1951

***P.picicornis* (Fabricius, 1793)**

**Material examined.** 1 ♀.

**Distribution in Georgia.** Sokhumi Botanical Garden (Danka 1950, 1957, 1968), Kintrishi National Park.

**Distribution in Caucasus.** Georgia (Danka 1950, 1957, 1968); North Caucasus: Karachay-Cherkessia (Golub 2016), Krasnodar Krai (Danka 1960, Golub 2016).

**Global distribution.** Holarctic (Lienhard 2016).

**Genetics.** We obtained a single barcode from the specimen with CaBOL-ID 1027558 (BOLD:ACB7137) identical to the COI of the *P.picicornis* from Canada (BOLD:ACB7137) in BOLD systems.

**Family Psocidae Roesler, 1940**

**Note.** Nine species are recorded from Georgia.

#### ﻿Genus *Amphigerontia* Kolbe, 1880

***A.contaminata* (Stephens, 1836)**

**Material examined.** 16 ♀♀, 19 ♂♂.

**Distribution in Georgia.** Sokhumi Botanical Garden (Danka 1950, 1957, 1968), Gori, Shilda, Tbilisi.

**Distribution in Caucasus.** Georgia (Danka 1950, 1957, 1968).

**Global distribution.** Holarctic (Lienhard 2016).

**Genetics.** We obtained nine barcodes from the specimens with CaBOL-IDs 1027554, 1027575, 1027576, 1027577, 1027631, 1033252, 1033253, 1033254 (BOLD:ACN1512, mean p-distance 0.89%) with the nearest neighbor in BOLD systems being *A.contaminata* from Germany with a private status (mean p-distance 1.14%).

#### ﻿Genus *Loensia Enderlein, 1924

* ***L.fasciata* (Fabricius, 1787)**

**Material examined.** 1♀, 1♂, 2 nymphs

**Distribution in Georgia.** Kintrishi National Park.

**Distribution in Caucasus.** Georgia.

**Global distribution.** Holarctic (Lienhard 2016).

**Genetics.** We obtained two barcodes from the specimens with CaBOL-IDs 1012705 and 1012878 (BOLD:ACP8983, p-distance 1.27%) with the nearest neighbor in BOLD systems being *L.fasciata* from Norway (BOLD:ACP8983, maximum p-distance 1.27%).

* ***L.variegata* (Fabricius, 1787)**

**Material examined.** 2 nymphs

**Distribution in Georgia.** Lebarde.

**Distribution in Caucasus.** Karachay-Cherkessia (Golub 2016), Georgia.

**Global distribution.** Palaearctic (Lienhard 2016).

**Genetics.** We obtained two identical barcodes from the specimens with CaBOL-IDs 1027655 and 1027656 (BOLD:ACN0757) with the nearest neighbor in BOLD systems being *L.variegata* from Finland (BOLD:ACN0757, mean p-distance 0.61%).

#### ﻿Genus *Neopsocus Kolbe, 1882

* ***N.rhenanus* Kolbe, 1882**

**Material examined.** 3 ♀♀ (brachypterous), 3 ♂♂.

**Distribution in Georgia.** Gori, Vardzia.

**Distribution in Caucasus.** Georgia.

**Global distribution.** European part of Western Palaearctic (Lienhard 2016).

**Genetics.** The three identical barcodes obtained from the specimens with CaBOL-IDs 1028013, 1028014, 1028015 (BOLD:AEV8540) are the first ones of the species submitted in BOLD systems.

**Remarks.** The species is characterized by brachypterous (short-winged) females and winged males.

#### ﻿Genus *Metylophorus Pearman, 1932

* ***M.nebulosus* (Stephens, 1836)**

**Material examined.** 10 ♀♀, 3 ♂♂.

**Distribution in Georgia.** Chkhorotsku, Churia, Kintrishi National Park.

**Distribution in Caucasus.** North Caucasus: Karachay-Cherkessia (Golub 2016), Krasnodar Krai (Danka 1960; Golub 2016); Georgia.

**Global distribution.** Palaearctic (Lienhard 2016).

**Genetics.** We obtained seven barcodes from the specimens with CaBOL-IDs 1012683, 1012875, 1030867, 1030868, 1033177, 1033178, 1033180 (BOLD:ADA4803, maximum p-distance 0.61%) with the nearest neighbor in BOLD systems being *Metylophorus* sp. from Russia (BOLD:ADA4803, mean p-distance 0.31%).

#### ﻿Genus *Psococerastis Pearman, 1932

* ***P.gibbosa* (Sulzer, 1776)**

**Material examined.** 10 ♀♀, 18 ♂♂.

**Distribution in Georgia.** Kintrishi National Park.

**Distribution in Caucasus.** North Caucasus: Karachay-Cherkessia (Golub 2016), Krasnodar Krai (Danka 1960; Golub 2016); Georgia.

**Global distribution.** Palaearctic (Lienhard 2016).

**Genetics.** Six barcodes were obtained from specimens with CaBOL-IDs 1012680, 1012681, 1012682, 1012733, 1012862 and 1012876 (BOLD:ACC5474). The newly obtained barcodes were nearly identical (maximum p-distance 0.15%) with the nearest neighbor in BOLD systems being *P.gibbosa* from Finland (BOLD:ACC5474, maximum p-distance 0.15%).

#### ﻿Genus *Psocus Latreille, 1794

* ***P.bipunctatus* (Linnaeus, 1761)**

**Material examined.** 3 ♂♂.

**Distribution in Georgia.** Kintrishi National Park, Mukhura.

**Distribution in Caucasus.** Georgia.

**Global distribution.** Palaearctic (Lienhard 2016).

**Genetics.** We obtained two barcodes from the specimens with CaBOL-IDs 1032272, 1032273 (BOLD:AFC1275, p-distance 0.76%) with the nearest neighbor in BOLD systems being *P.bipunctatus* from Finland (BOLD:ADD9199, mean p-distance 5.81%).

#### ﻿Genus *Trichadenotecnum Enderlein, 1909

* ***T.alexanderae* Sommerman, 1948**

**Material examined.** 6 ♀♀, 2 ♂♂.

**Distribution in Georgia.** Kintrishi National Park, Torsa.

**Distribution in Caucasus.** Georgia.

**Global distribution.** Nearctic (Lienhard 2016); Georgia.

**Genetics.** Four barcodes were obtained from specimens with CaBOL-IDs 1012703, 1012704, 1012837 and 1012838 (BOLD:ACE6318). The newly obtained barcodes were identical to each other as well as the nearest neighbor in BOLD systems *T.alexanderae* complex from Canada (BOLD:ACE6318).

**Remarks.** The only species from *T.alexanderae* complex known to occur in the Western Palaearctic is *T.innuptum* Betz, 1983 (Lienhard 2016), as we report *T.alexanderae* from this region for the first time.

* ***T.sexpunctatum* (Linnaeus, 1768)**

Fig. 4C

**Material examined.** 5 ♀♀, 4 ♂♂.

**Distribution in Georgia.** Telovani.

**Distribution in Caucasus.** North Caucasus: Karachay-Cherkessia (Golub 2016); Georgia.

**Global distribution.** Palaearctic (Lienhard 2016).

**Genetics.** Seven identical barcodes were obtained from specimens with CaBOL-IDs 1027295, 1027296, 1027298, 1027299, 1027300, 1027301 and 1027307 (BOLD:AFC4535) with the nearest neighbor in BOLD systems *T.sexpunctatum* from Finland with a private status (p-distance 1.53%).

**Family Stenopsocidae Kolbe, 1880**

**Note.** Three species are recorded from Georgia.

#### ﻿Genus *Enderleinella Badonnel, 1932

* ***E.obsoleta* (Stephens, 1836)**

**Material examined.** 2 ♀♀, 8 ♂♂.

**Distribution in Georgia.** Kintrishi National Park.

**Distribution in Caucasus.** Georgia.

**Global distribution.** European part of Western Palaearctic (Lienhard 2016).

**Genetics.** Two nearly identical barcodes were obtained from the specimens with CaBOL-IDs 1012706, 1027608 (BOLD:ACC7281, p-distance 0.15%) were identical to COI of *E.obsoleta* from Germany (BOLD:ACC7281).

#### ﻿Genus *Graphopsocus* Kolbe, 1880

***G.cruciatus* (Linnaeus, 1768)**

Fig. 4D

**Material examined.** 42 ♀♀, 212 ♂♂.

**Distribution in Georgia.** Batumi Botanical Garden (Danka 1955, 1957, 1968), Chkhorotsku, Churia, Gori, Kintrishi National Park, Khando, Sameba village (Kumisi vicinity), Shilda, Tbilisi.

**Distribution in Caucasus.** Armenia (Golub 2019), Georgia (Danka 1955, 1957, 1968), North Caucasus: Karachay-Cherkessia (Golub 2016), Krasnodar Krai (Danka 1960; Golub 2016).

**Global distribution.** Holarctic (Lienhard 2016).

**Genetics.** We obtained eight barcodes from the specimens with CaBOL-IDs 1012644, 1012645, 1012646, 1012647, 1012899, 1026995, 1030869, 1030880 (BOLD:ACA2933, mean p-distance 0.7%). The nearest neighbors in BOLD systems are as follows: 1012644, 1012645 (BOLD:ACA2933) to COI of *G.cruciatus* from Georgia (BOLD:AAF3894, similarity % = 100; 100 respectively); 1012646 (BOLD:ACA2933) to COI of *G.cruciatus* from Canada (BOLD:ACA2933, p-distance 0.31%); 1012647 (BOLD:ACA2933) to COI of *G.cruciatus* from Belarus (BOLD:AAF3894, p-distance 99.84); 1012899, 1026995, 1030869, 1030880 (BOLD:ACA2933) to COI of *G.cruciatus* from Norway (BOLD:ACA2933, similarity % = 98.31).

#### ﻿Genus *Stenopsocus* Hagen, 1866

***S.immaculatus* (Stephens, 1836)**

**Material examined.** 87 ♀♀, 60 ♂♂.

**Distribution in Georgia.** Batumi Botanical Garden (Danka 1955, 1957, 1968), Lagodekhi National Park, Kintrishi National Park.

**Distribution in Caucasus.** Georgia (Danka 1955, 1957, 1968).

**Global distribution.** Palaearctic (Lienhard 2016).

**Genetics.** Eight nearly identical barcodes were obtained from specimens with CaBOL-IDs 1012654, 1012655, 1012656, 1012657, 1012658, 1012725, 1012726 and 1012732 (BOLD:ABA6547, maximum p-distance 0.16%) with the nearest neighbor in BOLD systems being *S.immaculatus* from Finland (BOLD:ABA6547, mean p-distance 0.44%).

**Family Trichopsocidae Pearman, 1936**

**Note.** One species is recorded from Georgia.

#### ﻿Genus *Trichopsocus* Kolbe, 1882

***T.dalii* (McLachlan, 1867)**

**Material examined.** 38 ♀♀, 74 ♂♂.

**Distribution in Georgia.** Sokhumi Botanical Garden (Danka 1950, 1957, 1968), Batumi Botanical Garden (Danka 1955, 1957, 1968), Shilda, Tbilisi.

**Distribution in Caucasus.** Georgia (Danka 1950, 1955, 1957, 1968; as *Trichopsocuskolosvaryi* Danka, 1950), North Caucasus: Krasnodar KraaDanka 1950, 1960, 1968).

**Global distribution.** Holarctic (Lienhard 2016).

**Genetics.** We obtained six identical barcodes from the specimens with CaBOL-IDs 1027561, 1027562, 1027563, 1027567, 1027589, 1027590 (BOLD:AAP2620) with the nearest neighbor in BOLD systems being *T.dalii* from Canada (BOLD:AAP2620, mean p-distance 0.16%).

**Suborder Troctomorpha Roesler, 1940**

**Family Liposcelididae Enderlein, 1911**

**Note.** Four species are recorded from Georgia.

#### ﻿Genus *Embidopsocus Hagen. 1866

* ***E.enderleini* (Ribaga, 1905)**

**Material examined.** 1 ♀.

**Distribution in Georgia.** Shilda.

**Distribution in Caucasus.** Georgia.

**Global distribution.** Argentina; Austria; Bahrein; Belgium; France; Great Britain; Italy; Madeira; South Africa (Lienhard 2016); Croatia (Kučerova and Kalinović 2010).

**Remarks.** Described from Italy under the name of *Stenotroctesenderleini* (Ribaga 1905), *E.enderleini* belongs to the genus, which is most diversified in the South American-African region (Lienhard 2016). The species is characterized by winged females and apterous males, which mainly are found under the tree or shrub bark (Lienhard 1998).

#### ﻿Genus *Liposcelis* Motschulsky, 1852

* ***L.rufa* Broadhead, 1950**

**Material examined.** 7 specimens.

**Distribution in Georgia.** Tbilisi (in balcony crevices).

**Distribution in Caucasus.** Georgia.

**Global distribution.** Cosmopolitan.

**Genetics.** We obtained three virtually identical barcodes from the specimens with CaBOL-IDs 1027271, 1027272 and 1027275 (BOLD:ACW0584). The newly obtained barcodes were identical to the nearest neighbor in BOLD systems *L.rufa* from unknown place of origin (BOLD:ACW0584) mined from GenBank.

**Remarks.** This is a species with originally a Mediterranean native range (Schneider 2010)

* ***L.meridionalis* (Rosen, 1911)**

**Material examined.** 28 specimens.

**Distribution in Georgia.** Pia, Tbilisi, Vardzia.

**Distribution in Caucasus.** Armenia (Svadjan et al. 1963; Golub 2019), Georgia.

**Global distribution.** Western Palaearctic (Lienhard 2016).

**Genetics.** We obtained five nearly identical barcodes from the specimens with CaBOL-IDs 1030806, 1030807, 1030808, 1030810 (BOLD:AFC9720), 1030815 (BOLD:AFB9104) (maximum p-distance 0.76%). There are no barcodes of the species available in BOLD systems as we submit the first ones.

**Remarks.** Species with bisexual reproduction mainly occurring in the Mediterranean region, living in the litter, under the bark of trees, sometimes under stones (Lienhard 1977).

**Suborder Trogiomoropha Roesler, 1940**

Family *Psyllipsocidae Kolbe, 1884

**Note.** The representatives of the family have not been previously known to occur in Georgia. Two species have been recorded within the current study.

#### ﻿Genus *Dorypteryx Aaron, 1883

* ***D.domestica* (Smithers, 1958)**

**Material examined.** 1 ♀.

**Distribution in Georgia.** Batsara Strict Nature Reserve.

**Distribution in Caucasus.** Georgia.

**Global distribution.** Western Palaearctic; Zimbabwe (Lienhard 2016).

**Genetics.** A single barcode obtained from the specimen with CaBOL-ID 1027292 (BOLD:ACV6564) was identical to the nearest neighbor in BOLD systems *D.domestica* from Canada, France, and United States (BOLD:ACV6564).

**Remarks.***Dorypteryxdomestica* originates from Africa (Schneider 2010), with the first record in Europe from Switzerland (Lienhard 1977).

#### ﻿Genus *Psyllipsocus Selys-Longchamps, 1872

* ***P.ramburii* Selys-Longchamps, 1872**

**Material examined.** 2 ♀♀, micropterous.

**Distribution in Georgia.** Gori.

**Distribution in Caucasus.** Armenia (Svadjan et al. 1963; Danka 1968; Golub 2019), Georgia.

**Global distribution.** Cosmopolitan (Lienhard 2016).

**Remarks.** Cryptogenic species with unknown native range (Schneider 2010).

Family *Trogiidae Enderlein, 1911

**Note.** The representatives of the family have not been previously known to occur in Georgia. Two species have been recorded within the current study.

#### ﻿Genus *Cerobasis* Kolbe, 1882

* ***C.guestfalica* (Kolbe, 1880)**

**Material examined.** 4 ♀♀.

**Distribution in Georgia.** Grigoleti, Qvabiskhevi.

**Distribution in Caucasus.** Georgia.

**Global distribution.** Cosmopolitan (Lienhard 2016).

**Remarks.***Cerobasisguestfalica* generally reproduces via parthenogenesis. Males are known from Great Britain and Poland (Lienhard 1984) and have also been observed in Germany (Nicolai 1985, 1987). Cases of ovoviviparity have been reported by Jentsch 1936. The species is often abundant on the bark of various trees and bushes, but it also lives in the herbaceous layer or in leaf litter. Females are apterous.

#### ﻿Genus *Lepinotus Heyden, 1850

* ***L.reticulatus* (Linnaeus, 1768)**

**Material examined.** 25 ♀♀.

**Distribution in Georgia.** Dighomi village, Gori, Pia, Tbilisi, Vardzia.

**Distribution in Caucasus.** Armenia (Golub 2019), Azerbaijan (Danka 1968), Georgia. Indication of the species presence in Georgia by Khandehroo et al. (2015) is erroneous.

**Global distribution.** Cosmopolitan (Lienhard 2016).

**Genetics.** We obtained five nearly identical barcodes from the specimens with CaBOL-IDs 1030801, 1030802, 1030803, 1030804, 1030805 (BOLD:AFC5414, maximum p-distance 0.61%) with the nearest neighbor in BOLD systems being *L.reticulatus* from Canada (BOLD:ADD4959, mean p-distance 1.57%).

**Remarks.** Cryptogenic species of unknown origin (Schneider 2010). Mainly found in buildings with food storages in Central Europe (Schneider 2010; Svensson and Hall 2010) and the south, especially in the Mediterranean, where it is found in more natural places, like dried grass or leaf litter (Lienhard 1998). *Lepinotusreticulatus* is parthenogenetic and so there are only females, although a few times (non-functional) males have been reported (Lienhard 1998).

## ﻿Discussion

Danka laid a profound basis with her research on the Psocoptera fauna of Georgia, even though it was limited to only a few locations almost exclusively in Batumi and Sokhumi Gardens located at the Black Sea coast. The material collected and examined in our study mostly also originates from the western part of Georgia (see Suppl. material 1 and Fig. 1) and had more of a bycatch character than a purposeful collection of Psocoptera, but still resulted in 31 species, three families (Amphipcosidae, Elipsocidae, Psyllipsocidae) and one suborder (Trogiomorpha) that have never been previously reported from the country. Of 31 species reported for the first time from Georgia, 24 have been known to occur in the adjacent countries, 37 from the European part of the post-Soviet space and six (*Embidopsocusenderleini*, *Dorypteryxdomestica*, *Cerobasisguestfalica*, *Liposcelisrufa*, *Ectopsocusmeridionalis and Trichadenotecnumalexanderae*) have never been reported from the territories mentioned above (Table 2). Lack of specialists and targeted studies from other parts of the country on this particular group of minuscule animals has left many more species undiscovered/undescribed. After analyzing the fauna of neighboring countries of Georgia, as well as countries of the Western Palaearctic European part of the post-Soviet space (Table 2) there are at least 14 species still expected to be found in Georgia. New taxa could also be discovered in unexplored areas such as for instance caves (Lienhard 2021) and other underground habitat, thus the psocid fauna of Georgia still remains understudied.

**Table 2.** Census on Psocoptera of Georgia. ^1^Classification according Johnson et al. (2022).

Taxon name^1^	Species reported from Georgia		Species reported from ex-USSR Western Palaearctic European part (Lienhard 2006; 2016)	Species reported from adjacent countries				Estimated species unrecorded from Georgia
	Danka (1968)	Current study		Karachaevo-Cherkessia (Golub 2016)	Armenia (Golub 2019; Lienhard 2021)	Azerbaijan (Danka 1968)	Turkey (Lienhard 2005, 2016; Ozsisli 2010)	
**Suborder Psocomorpha**								
**Amphipsocidae**								
*Kolbiaquisquiliarum*		+	+					
**Caeciliusidae**								
*Caeciliusfuscopterus*		+	+	+	+			
*Valenzuelalabinae*			+					
*Valenzuelaatricornis*		+	+					
*Valenzuelaburmeisteri*	+	+	+	+				
*Valenzuelacorsicus*							+	
*Valenzueladespaxi*			+	+				+
*Valenzuelaflavidus*	+	+	+	+	+		+	
*Valenzuelagynapterus*			+					
*Valenzuelapiceus*	+	+	+	+				
**Ectopsocidae**								
*Ectopsocopsiscryptomeriae*		+	+					
*Ectopsocusbriggsi*	+	+	+		+			
*Ectopsocusmeridionalis*		+						
*Ectopsocus* spec.			+					
*Ectopsocusvishnyakovae*		+	+		+			
*Ectopsocopsisxerophylla*			+					
**Elipsocidae**								
*Cuneopalpuscyanops*			+					
*Elipsocusabdominalis*			+					
*Elipsocushyalinus*		+	+					
*Elipsocuspumilis*			+					
*Elipsocusmoebiusi*		+		+	+		+	
*Hemineuradispar*			+				+	+
*Hemineurahispanica*		+	+		+			
*Pseudopsocusfusciceps*			+					
*Reuterellahelvimacula*			+				+	+
**Epipsocidae**								
*Bertkauialucifuga*		+	+				+	+
**Lachesillidae**								
*Lachesilla* spec.			+					
*Lachesillabernardi*		+					+	
*Lachesilladimorpha*							+	
*Lachesillapedicularia*		+	+		+			
*Lachesillaquercus*	+	+	+	+	+		+	
*Lachesillarossica*			+					
*Lachesillatanaidana*			+					
**Mesopsocidae**								
*Mesopsocusimmunis*			+					
*Mesopsocuslaticeps*		+	+					
*Mesopsocusunipunctatus*	+		+	+	+			
*Mesopsocusvernus*							+	
**Peripsocidae**								
*Peripsocusalboguttatus*	+	+	+	+			+	
*Peripsocusdidymus*		+	+					
*Peripsocusgolubae*		+	+					
*Peripsocusparvulus*			+	+	+			+
*Peripsocusphaeopterus*	+	+	+	+			+	
*Peripsocussubfasciatus*	+	+	+	+				
**Philotarsidae**								
*Aaroniellabadonneli*	+	+	+					
*Philotarsuspicicornis*	+	+	+					
**Psocidae**								
*Amphigerontiabifasciata*			+	+				+
*Amphigerontiacontaminata*	+	+	+					
*Amphigerontiaintermedia*			+					
*Blasteconspurcata*			+	+			+	+
*Blastequadrimaculata*			+					
*Hyalopsocuscontrarius*			+	+				+
*Hyalopsocusmorio*			+					
*Loensiafasciata*		+	+					
*Loensiapearmani*			+					
*Loensiavariegata*		+	+	+				
*Metylophorusnebulosus*		+	+	+				
*Neopsocopsishirticornis*			+					
*Neopsocusrhenanus*		+	+				+	
*Psocidusflavonimbatus*			+					
*Psococerastisgibbosa*		+	+	+				
*Psocusbipunctatus*		+	+					
*Trichadenotecnumalexanderae*		+						
*Trichadenotecnumgermanicum*			+					
*Trichadenotecnummajus*			+	+				+
*Trichadenotecnumsexpunctatum*		+	+	+				
**Stenopsocidae**								
*Enderleinellaobsoleta*		+	+					
*Graphopsocuscruciatus*	+	+	+	+	+		+	
*Stenopsocusimmaculatus*	+	+	+					
*Stenopsocuslachlani*			+	+				+
*Stenopsocusstigmaticus*			+					
**Trichopsocidae**								
*Trichopsocusclarus*			+					
*Trichopsocusdalii*	+	+	+					
**Suborder Troctomorpha**								
**Liposcelididae**								
*Embidopsocusenderleini*		+						
*Liposcelisbostrychophila*							+	+
*Liposcelisbrunnea*			+					
*Liposcelisdecolor*	+						+	
*Liposcelisdivinatoria*			+		+			+
*Liposcelisentomophila*							+	
*Liposcelisformicaria*			+					
*Liposcelismeridionalis*		+	+		+			
*Liposcelissilvarum*			+		+			+
*Liposcelisrufa*		+						
*Liposcelistricolor*							+	
**Protroctopsocidae**								
*Reticulopsocusbesucheti*							+	
**Suborder Trogiomorpha**								
**Prionoglarididae**								+
*Prionoglariskapralovi*					+			
*Prionoglarisstygia*							+	
**Psyllipsocidae**								
*Dorypteryxdomestica*		+						
*Psyllipsocusramburii*		+	+		+			
**Trogiidae**								
*Cerobasisannulata*			+					
*Cerbosasguestfalica*		+						
*Lepinotusinquilinus*			+					
*Lepinotusreticulatus*		+	+		+	+	+	
*Trogiumpulsatorium*			+					

This study, originally conceived as a small addition to the fauna of Georgian psocopterans, eventually turned into a large-scale exciting journey through the study of a new group of arthropods for the corresponding author. In the end, we hope that our study inspires the readership to spend more time in nature observing and unveiling the hidden gems, reminding them that science begins with subsiding the mind’s curiosity.

## ﻿Additional information

### Author ORCIDs

Armen Seropian https://orcid.org/0000-0003-3777-9954

Eka Arsenashvili https://orcid.org/0000-0002-7330-9602

Natalia Bulbulashvili https://orcid.org/0000-0002-6802-1209

Anano Shubashishvili https://orcid.org/0000-0002-9747-2703

Giorgi Iankoshvili https://orcid.org/0000-0002-0429-1488

Mariami Todua https://orcid.org/0000-0003-1870-3030

Ana Ananiashvili https://orcid.org/0000-0002-4186-0723

Shota Japarashvili https://orcid.org/0000-0002-9674-3719

Tinatin Chkhartisvhili https://orcid.org/0000-0002-0429-1488

Aleksi Memishishi https://orcid.org/0000-0002-5862-5105

Sopio Balkhamishvili https://orcid.org/0000-0001-5198-305X

Beka Chitadze https://orcid.org/0000-0001-7311-5827

Elisabeth Karalashvili https://orcid.org/0000-0002-9015-7604

Levan Mumladze https://orcid.org/0000-0002-2172-6973

Nils Hein https://orcid.org/0000-0002-5172-8531

Björn Rulik https://orcid.org/0000-0002-2808-9305

### Data availability

All of the data that support the findings of this study are available in the main text or Supplementary Information.

## Supplementary materials

Supplementary material 1 Details for sampling locations Data type excel file https://binary.pensoft.net/file/869418This dataset is made available under the Open Database License (http://opendatacommons.org/licenses/odbl/1.0/). The Open Database License (ODbL) is a license agreement intended to allow users to freely share, modify, and use this Dataset while maintaining this same freedom for others, provided that the original source and author(s) are credited.Armen Seropian, Eka Arsenashvili, Natalia Bulbulashvili, Anano Shubashishvili, Giorgi Iankoshvili, Mariami Todua, Ana Ananiashvili, Shota Japarashvili, Tinatin Chkhartisvhili, Aleksi Memishishi, Sopio Balkhamishvili, Beka Chitadze, Elisabeth Karalashvili, Levan Mumladze, Nils Hein, Björn Rulik

## References

[B1] Anonby JE (2019) Psocoptera of Canada. In: Langor DW Sheffield CS (Eds) The Biota of Canada – A Biodiversity Assessment. Part 1: The Terrestrial Arthropods.ZooKeys 819: 295–299. 10.3897/zookeys.819.27640

[B2] Astrin JJ Stüben PE (2008) Phylogeny in cryptic weevils: molecules, morphology and new genera of western Palaearctic Cryptorhynchinae (Coleoptera: Curculionidae). Invertebrate Systematics 22(5): 503–522. 10.1071/IS07057

[B3] Danka L (1950) Psocoptera of Sokhumi Botanical Garden. Soobshchenie Gosudarstvennogo Muzeja Prirody, Riga 2: 1–3. [In Russian]

[B4] Danka L (1955) Psocoptera of the Batumi and Sochi Botanical Gardens. Entomologicheskoe Obozrenie 34: 180–184. [In Russian]

[B5] Danka L (1957) Psocoptera of Georgia. Theses of Addresses of the 3^rd^ Congress of the All-Union Entomological Society. Izdatelstwo Akademii Nauk SSSR. Leningrad. (Psocoptera: 7–8). [In Russian]

[B6] Danka L (1960) Jaunas zinas par Padomju Savienibas kerpjutu faunu. Latvijas Entomologs 1: 29–33.

[B7] Danka L (1968) Catalogue of the Psocoptera of the USSR. Latvijas Entomologs 12: 3–18. [In Russian]

[B8] de Moya RS (2022) Illumina whole genome sequencing indicates ploidy level differences within the *Valenzuelaflavidus* (Psocodea: Psocomorpha: Caeciliusidae) species complex. Systematic Entomology 47(1): 202–212. 10.1111/syen.12525

[B9] Golub NV (2016) New records of bark-lice (Psocoptera) for the Karachay-Cherkess Republic (Russia). Evraziatskii Entomologicheskii Zhurnal 15(2): 183–187. [in Russian]

[B10] Golub NV (2019) The bark-lice (Psocoptera) fauna of the Republic of Armenia. Evraziatskii Entomologicheskii Zhurnal 18(3): 196–198. 10.15298/euroasentj.18.3.09 [in Russian]

[B11] Jentsch S (1936) Ovoviviparie bei einer einheimischen Copeognathenart (*Hyperetesguestphalicus*). Zoologischer Anzeiger 116: 287–289.

[B12] Johnson KP Smith VS Hopkins HH (2022) Psocodea Species File Online. Version 5.0/5.0. http://Psocodea.SpeciesFile.org [Accessed on 17 July 2022]

[B13] Khandehroo F Gholamhossein M Sadeghi H Fekrat L (2015) First report of *Lepinotusreticulatus* and *Ectopsocusvishnyakovae* (Insecta: Psocoptera) from Iran. Journal of Entomological Society of Iran 35: 73–74.

[B14] Kučerova Z Kalinović I (2010) First Record of *Embidopsocusenderleini* (Ribaga, 1905) (Psocoptera: Liposcelididae) for Croatia. Entomologia Croatica 14(1–2): 135–138.

[B15] Lienhard C (1977) Die Psocopteren des schweizerischen Nationalparks und seiner Umgebung (Insecta: Psocoptera). Ergebnisse der wissenschaftlichen Untersuchungen im Schweizerischen Nationalpark 14: 417–551. 10.3929/ethz-a-000091744

[B16] Lienhard C (1984) Etudes préliminaires pour une faune des Psocoptères de la région ouestpaléarctique. I. Le genre *Cerobasis* Kolbe, 1882 (Psocoptera: Trogiidae). Revue Suisse de Zoologie 91: 747–764. 10.5962/bhl.part.81579

[B17] Lienhard C (1998) Psocoptères euro-méditerranéens. Faune de France 83: 1–517.

[B18] Lienhard C (2005) Description of a new beetle-like psocid (Insecta: Psocoptera: Protroctopsocidae) from Turkey showing an unusual sexual dimorphism. Revue Suisse de Zoologie 112(2): 333–350. 10.5962/bhl.part.80302

[B19] Lienhard C (2006) Four interesting psocids (Psocodea:‘Psocoptera’) from European parts of Russia and from the eastern Mediterranean. Revue Suisse de Zoologie 113(4): 807–816. 10.5962/bhl.part.80377

[B20] Lienhard C (2016) Country checklists of the Psocoptera species of the World, extracted from Lienhard & Smithers, 2002: Psocoptera (Insecta) - World Catalogue and Bibliography. Psocid News 1: 1–123.

[B21] Lienhard C (2021) A new species of *Prionoglaris* Enderlein (Psocodea: ‘Psocoptera’: Prionoglarididae) from an Armenian cave, with an account of the distribution of the genus. Revue Suisse de Zoologie 128(2): 227–235. 10.35929/RSZ.0048

[B22] Lyal CHC (1985) Phylogeny and classification of the Psocodea, with particular reference to the lice (Psocodea: Phthiraptera). Systematic Entomology 10: 145–165. 10.1111/j.1365-3113.1985.tb00525.x

[B23] Mockford EL (1993) North American Psocoptera (Insecta). Sandhill Crane Press, Gainesville, Florida, Flora and Fauna Handbook 10, [xviii +] 455 pp.

[B24] Nicolai V (1985) Die ökologische Bedeutung verschiedener Rindentypen bei Bäumen. Diss. Univ. Marburg, 198 pp.

[B25] Nicolai V (1987) Anpassungen rindenbesiedelnder Arthropoden an Borkenstruktur und Feinddruck. Spixiana 10(2): 139–146.

[B26] Ozsisli T (2010) First record for Turkish fauna: *Liposcelisbostrychophila* Badonnel, 1931 (Psocoptera: Liposcelididae). Turkiye Entomoloji Dergisi 34: 379–382.

[B27] Ratnasingham S Hebert PDN (2013) A DNA-based registry for all animal species: The Barcode Index Number (BIN) system. PLoS ONE 8(7): e66213. 10.1371/journal.pone.0066213 PMC3704603 23861743

[B28] Saville B (2008) National Barkfly Recording Scheme (Britain and Ireland). https://www.brc.ac.uk [Accessed on 17 July 2022]

[B29] Schneider N (2010) Psocids (Psocoptera). In: Roques A Kenis M Lees D Lopez-Vaamonde C Rabitsch J-Y Roy DB (Eds) Alien terrestrial arthropods of Europe.BioRisk 4(2), 793–805. 10.3897/biorisk.4.46

[B30] Svadjan PK Vishnyakova VN Marjanjan KS (1963) To the fauna of the Psocoptera of Armenian SSR and the methodology of their laboratory cultivation. Izvestiya Akademii Nauk Armyanskoj SSR: Biologicheskie nauki 16(9): 88–94. [In Russian]

[B31] Svensson B Hall K (2010) Nationalnyckeln till Sveriges flora och fauna. Stövsländor. Psocoptera. ArtDatabanken, SLU, Uppsala, 204 pp. [+ numerous figs]

[B32] Thormann J Ahrens D Anderson C Astrin JJ Mumladze L Rulik B Tarkhnishvili D Espeland M Geiger M Hein N Iankoshvili G Karalashvili E Mengual X Morkel C Neiber MT Peters RS Reimann A Ssymank A Wesener T Ziegler J Misof B (2019) A prelude to the Caucasus Barcode of Life Platform (CaBOL): Biodiversity Days in Georgia in 2018 and 2019. Bonn Zoological Bulletin 68: 275–296.

[B33] Yoshizawa K Lienhard C (2010) In search of the sister group of the true lice: a systematic review of booklice and their relatives, with an updated checklist of Liposcelididae (Insecta: Psocodea). Arthropod Systematics & Phylogeny 68: 181–195.

